# Ubiquitin-Mediated Regulation of Endocytosis by Proteins of the Arrestin Family

**DOI:** 10.1155/2012/242764

**Published:** 2012-09-04

**Authors:** Michel Becuwe, Antonio Herrador, Rosine Haguenauer-Tsapis, Olivier Vincent, Sébastien Léon

**Affiliations:** ^1^Institut Jacques Monod, Centre National de la Recherche Scientifique, UMR 7592, Université Paris Diderot, Sorbonne Paris Cité, 75205 Paris, France; ^2^Instituto de Investigaciones Biomédicas, CSIC-UAM, Arturo Duperier, 4, 28029 Madrid, Spain

## Abstract

In metazoans, proteins of the arrestin family are key players of G-protein-coupled receptors (GPCRS) signaling and trafficking. Following stimulation, activated receptors are phosphorylated, thus allowing the binding of arrestins and hence an “arrest” of receptor signaling. Arrestins act by uncoupling receptors from G proteins and contribute to the recruitment of endocytic proteins, such as clathrin, to direct receptor trafficking into the endocytic pathway. Arrestins also serve as adaptor proteins by promoting the recruitment of ubiquitin ligases and participate in the agonist-induced ubiquitylation of receptors, known to have impact on their subcellular localization and stability. Recently, the arrestin family has expanded following the discovery of arrestin-related proteins in other eukaryotes such as yeasts or fungi. Surprisingly, most of these proteins are also involved in the ubiquitylation and endocytosis of plasma membrane proteins, thus suggesting that the role of arrestins as ubiquitin ligase adaptors is at the core of these proteins' functions. Importantly, arrestins are themselves ubiquitylated, and this modification is crucial for their function. In this paper, we discuss recent data on the intricate connections between arrestins and the ubiquitin pathway in the control of endocytosis.

## 1. Introduction

The name of “arrestin” was initially given to a 48-kDa protein that was essential to “arrest” the signal following the photoexcitation of rhodopsin, a photoreceptor of the G-protein-coupled receptors (GPCRS) family expressed in rod and cone cells of the retina [1, 2]. A second isoform involved in the same process has later been identified; both of these proteins are now designated visual arrestins (or arrestin-1 and -4) (for review see [3]). A similar regulatory system was described for another GPCR, the *β*2-adrenergic receptor (*β*2-AR), which involves two other arrestins, named *β*-arrestin-1 and -2 (or arrestin-2 and -3, resp.) [4–6]. *β*-arrestins are ubiquitously expressed and were later found to regulate a large number of receptors in addition to *β*2-AR.

## 2. Arrestin-Mediated Regulation of GPCRs

### 2.1. Arrestin-Dependent Uncoupling of GPCRs from G-Proteins

Arrestins are key players in the regulation of GPCR signaling activity. Upon agonist stimulation, GPCRs undergo conformational changes leading to their association to heterotrimeric G proteins and subsequent activation, thereby triggering appropriate signal transduction pathways. Receptor desensitization is initiated after ligand binding through the phosphorylation of residues within their cytosolic loops by G-protein-coupled receptor kinases (GRKs). This modification allows arrestin docking to the GPCR, which in turn favors the uncoupling between the receptor and the G protein. Indeed, *β*-arrestins are cytosolic proteins that, in response to receptor stimulation, relocalize rapidly to the plasma membrane [7]. Structural and structure-function studies of visual arrestins identified a phosphate-sensor domain in the polar core of the protein [8]. Intramolecular interactions between the C-terminal tail and the phosphate sensor region maintain the arrestin in an inactive state, and this interaction is disrupted upon binding to the phosphorylated receptor. This interaction is followed by a conformational change of the arrestin molecule and leads to a high-affinity receptor-binding state. Arrestin recruitment onto the phosphorylated receptor hinders its interaction with G protein, and consequently silences the activation of the GPCR-G protein-signaling module.

### 2.2. Arrestins and GPCR Endocytosis

Another crucial component of GPCR regulation operates at the level of their localization [9]. Endocytosis plays a major role in the modulation of GPCR signaling activity, and, again, this regulation involves *β*-arrestins [10]. Indeed, *β*-arrestins act as endocytic adaptor proteins that recruit components of the endocytosis machinery to promote GPCR internalization and/or degradation. *β*-arrestins interact with clathrin through a clathrin-binding motif [11–13] to promote GPCR association to clathrin-coated pits (CCPs). Deletion of the clathrin-binding site abrogates arrestin-promoted trafficking of the *β*2-AR [14]. Additionally, *β*-arrestins interact with the clathrin adaptor complex AP-2 upon GPCR binding [14–16], to promote clathrin-coat assembly and receptor targeting to CCPs [17]. In addition to clathrin, *β*-arrestins also bind to other components of the endocytic machinery such as the N-ethylmaleimide-sensitive fusion protein (NSF), the small G protein ARF6, and the phosphatidylinositol 4-phosphate 5 kinase PIP5 K I*α* [18–20].

### 2.3. Arrestins as Signaling Scaffolds

Besides their functions in GPCR desensitization and trafficking, *β*-arrestins are also capable of generating their own signals by scaffolding signaling molecules, such as non-receptor tyrosine kinases of the Src family, or MAP (mitogen-activated protein) kinases (ERK1/2, c-Jun N-terminal kinase 3 JNK3) (reviewed in [21]). *β*-arrestins therefore mediate a second wave of signaling distinct from G-protein-dependent signaling.

### 2.4. Arrestins and Ubiquitin

*β*-arrestins were also shown to regulate the final fate of the receptor, by acting on the balance between receptor recycling to the plasma membrane, or its lysosomal degradation. The posttranslational modification of plasma membrane proteins, including receptors, by ubiquitin is known to affect their sorting along the endocytic pathway [22, 23]. *β*-arrestins have the ability to recruit ubiquitin ligases and promote receptor ubiquitylation, therefore acting as “adaptor” proteins [24]. Interestingly, a phylogenetic study has revealed that proteins of the arrestin family are present in all eukaryotes, except plants [25, 26]. A body of evidence (detailed later in this review, [27]) indicates that these arrestin-related proteins are also involved in the regulation of plasma membrane proteins trafficking by acting as ubiquitin ligase adaptors. Therefore, this function seems to be one of the most conserved features within the arrestin family [28, 29]. 

Both arrestins and arrestin-related proteins are themselves targets of ubiquitylation. This was discovered very early on for *β*-Arr2 in response to agonist stimulation [30]. Likewise, the fungal arrestin-related protein PalF was shown to be ubiquitylated in response to alkaline ambient pH, in a signal- and receptor- (PalH) dependent manner [31]. This ubiquitylation appeared crucial for the proper function of arrestins [24, 32–35], but the precise role of this modification is poorly understood. An additional layer of complexity has recently been added following the observations that *β*-arrestins interact with deubiquitylating enzymes that regulate their ubiquitylation status as well as receptor ubiquitylation and, consequently, their fate [35–37].

In this review, we will focus on the connections between arrestins and ubiquitin. We will detail the function of arrestins and arrestin-related proteins as ubiquitin ligase adaptor and discuss how arrestin functions could be regulated by ubiquitylation.

## 3. Arrestins as Ubiquitin Ligase Adaptors

### 3.1. Ubiquitin and Endocytic Protein Sorting

Studies in the last decades have shown that ubiquitin is a master regulator of endocytosis in eukaryotes. Early work performed in the yeast *Saccharomyces cerevisiae *demonstrated that ubiquitin is involved in the endocytosis of plasma membrane proteins, such as ABC (ATP-binding cassette) transporters [38], receptor[39, 40], or permeases [41]. The ubiquitylation of plasma membrane proteins appears to trigger their internalization and targeting to endosomes [42], although the existence of an ubiquitin-independent internalization mechanism is also documented [43]. In mammalian cells, the situation is more complex, as several internalization pathways exist in the cell with only some of them regulated by ubiquitin [44].

Initially, it has been proposed that ubiquitylated cargoes are recognized in yeast and mammals by the ubiquitin-binding motifs of various proteins involved in endocytosis, such as Eps15 (Ede1 in yeast) and Epsin (Eps15 interacting; Ent1 and Ent2 in yeast) which display UIM (ubiquitin-interacting motif) or UBA (ubiquitin-associated) domains [45, 46]. In addition, these endocytic proteins can also interact with phosphoinositides and clathrin, making them ideal candidates to coordinate ubiquitin recognition and cargo internalization. While such a function appears to be established in mammalian cells [47–49], recent data in yeast favor a more complex model, where ubiquitin-binding domains would play a more general role in protein interactions and the assembly of the endocytic network [50]. Noteworthy, in mammalian cells, endocytic adaptors are often ubiquitylated in response to extracellular stimuli, and this contributes greatly to the ubiquitin-based signaling triggered upon cell stimulation [23, 51–53].

A second major ubiquitin-dependent step in the endocytic pathway occurs at multivesicular bodies (MVBs) and is required for cargo delivery into lysosomes [54]. Cargo ubiquitylation provides the crucial signal for entering into this pathway. A series of protein complexes, collectively named ESCRT (endosomal sorting complex required for transport) carry ubiquitin-binding domains and act in concert to allow the recognition and sorting of ubiquitylated cargoes into luminal vesicles of MVBs [55]. Therefore, lack of cargo ubiquitylation at this stage leads to a defective targeting to the lysosome, and, eventually, recycling [56].

In mammalian cells, initial studies showed that the ubiquitin conjugation system is important for the downregulation of the growth hormone receptor (GHR) [57]. Also, the study of the amiloride-sensitive epithelial sodium channel ENaC clearly established that its ubiquitylation regulates the channel's stability [58]. Subsequent work on ENaC, GHR, and many other receptors (such as EGFR, PDGFR, c-Met, TGF-*β*R, *β*2-AR) confirmed the critical function of ubiquitin in endocytosis in mammals [23, 59, 60]. However, where this ubiquitylation occurs in the cell (plasma membrane or endosomal compartments), and how ubiquitylation impacts on the target receptor's fate (internalization, progression through the endocytic pathway, or degradation) are still a matter of debate and seem to vary upon the receptor and the physiological situation considered [61]. Also, it should be noted that while ubiquitin-mediated endocytosis appears as the main pathway in yeast, ubiquitin-independent endocytosis is more represented in higher eukaryotes [44, 62]. 

### 3.2. The “Classic” *β*-Arr2/*β*2-AR Couple

A first evidence for the role of arrestins in receptor ubiquitylation came from a study by Shenoy and colleagues who observed that the *β*2-AR is ubiquitylated within 15 min of isoproterenol stimulation, ultimately leading to receptor degradation [30].*β*2-AR ubiquitylation requires *β*-Arr2 and the ubiquitin ligase MDM2, which turned out to ubiquitylate *β*-Arr2 rather than *β*2-AR (see below) [30]. A mutant *β*2-AR lacking the ubiquitylation sites (*β*2 − *AR* ^K0^) is normally internalized, but not degraded [30]. In contrast, a translational fusion of ubiquitin to the *β*2-AR, which mimics its constitutive ubiquitylation, is internalized similarly as the wild-type *β*2-AR, but is degraded more efficiently [24].

Therefore, ubiquitylation is a critical signal for *β*2-AR degradation upon stimulation. A similar implication of GPCR ubiquitylation in its degradation, but not in its internalization, was also reported in the case of CXCR4 [63]. The identity of the ubiquitin ligase responsible for *β*2-AR ubiquitylation was revealed more recently. Indeed, *β*-Arr2 was found to interact with the HECT-type (homologous to E6-AP C-terminus) ubiquitin ligase Nedd4 (discussed below in this paper). Nedd4 (neural precursor cell expressed developmentally downregulated protein 4) promotes *β*2-AR ubiquitylation at endosomes, leading to its lysosomal targeting [24].

Once internalized, GPCRs can also escape degradation and recycle back to the plasma membrane in a functional state to mediate further signaling. Because GPCR ubiquitylation appears to trigger its degradation, deubiquitylation could regulate GPCR recycling to the plasma membrane. Indeed, two deubiquitylating enzymes named USP33 and USP20 regulate *β*2-AR deubiquitylation, recycling and resensitization [35, 37]. USP33 was first identified as a *β*-arrestin interactant, thus suggesting that *β*-Arr2 could be involved in USP33 recruitment to *β*2-AR [35]. However, USP33 was found to interact with *β*2-AR even before agonist stimulation, that is, when *β*-Arr2 is not yet translocated to the plasma membrane [37]. In fact, USP33 appears to be transferred from agonist-activated *β*2-AR to *β*-Arr2, thus triggering its deubiquitylation and dissociation from the receptor, once internalized. Reassociation of USP33 with *β*2-AR in endosomal compartments would regulate its deubiquitylation and recycling to the plasma membrane. Thus, the association and dissociation of *β*-Arr2 from *β*2-AR may coordinate the ubiquitin conjugating/deconjugating activities towards *β*2-AR to tune the balance between receptor degradation and recycling. This positions the ubiquitin ligase adaptor function of *β*-Arr2 as a key regulator of GPCR signaling.

### 3.3. *β*-Arrestins as Ubiquitin Ligase Adaptors: Other Examples

The function of *β*-arrestins as ubiquitin ligase adaptors is not restricted to *β*2-AR. Additional studies identified *β*-arrestins as ubiquitin ligase adaptors for non-GPCR proteins: *β*-Arr1, as *β*-Arr2, acts as an adaptor for ubiquitin ligases of the Nedd4 family such as Itch/AIP4 (Atrophin-1-interacting protein 4) for ubiquitylation of the TRPV4 (transient receptor potential) channel [64], and Nedd4 for that of the Na^+^/H^+^ exchanger 1 (NHE1) [65]. In the latter case, however, and in contrast to the situation described for the *β*2-AR, cargo ubiquitylation is required for its internalization. Because *β*-arrestins interact with both ubiquitin ligase and clathrin (see above), they may then act at two levels: first, for cargo ubiquitylation, which could recruit Eps15/Epsin endocytic adaptors, and, second, to assist the latter in the recruitment of a clathrin coat.

The contribution of *β*-arrestins to the trafficking of another classical GPCR, the chemokine receptor, CXCR4, was also studied. Early reports had shown that the ligand-induced ubiquitylation of CXCR4 by the Nedd4-like ubiquitin ligase AIP4 is required for its lysosomal sorting [63, 66]. *β*-Arr1 interacts with AIP4 at endosomes, and knockdown experiments revealed that *β*-Arr1 is an important player in CXCR4 degradation but, surprisingly, is not required for its ubiquitylation [67]. Instead, CXCR4 is phosphorylated at the plasma membrane after ligand binding, which allows the direct recruitment of the ubiquitin ligase AIP4 via its WW domains, and hence CXCR4 ubiquitylation [68]. *β*-Arr1 was later found to interact with the ESCRT-0 complex and to direct the ubiquitylation of one of its components, HRS (hepatocyte growth factor-regulated tyrosine kinase substrate) in a CXCR4-dependent manner [69]. 

Interestingly, *β*-arrestins appear to act primarily as adaptors for ubiquitin ligase of the Nedd4 family. These enzymes display WW domains that can interact with specific proline-rich motifs (usually, a [L/P]PxY sequence). Although this motif is sometimes present on the targeted substrates, as in the case of ENaC [70], in most cases this interaction motif is present on an adaptor protein in charge of substrate recognition [71]. However, no PPxY motif has been found in *β*-arrestins, and polyproline regions are not involved in Nedd4 interaction [24]. In addition, Nedd4 recruitment to *β*2-AR was not affected by mutations in Nedd4 WW domains. This indicates that this interaction involves a noncanonical binding of *β*-Arr2 to Nedd4, for which the molecular determinants remain to be addressed.

In some cases, *β*-arrestins act as adaptors for ubiquitin ligase which do not belong to the Nedd4 family. *β*-Arr1 was proposed to act as an adaptor for the RING (Really interesting new gene) ubiquitin ligase Mdm2 to mediate insulin-like growth factor I (IGF-1) receptor ubiquitylation and downregulation [72, 73]. A similar role was appointed to *β*-Arr2 for the ubiquitylation of the androgen receptor [74]. Again, the molecular basis of this interaction awaits further investigations.

## 4. Arrestin-Related Proteins: New Players in the Field

**Figure 1:**
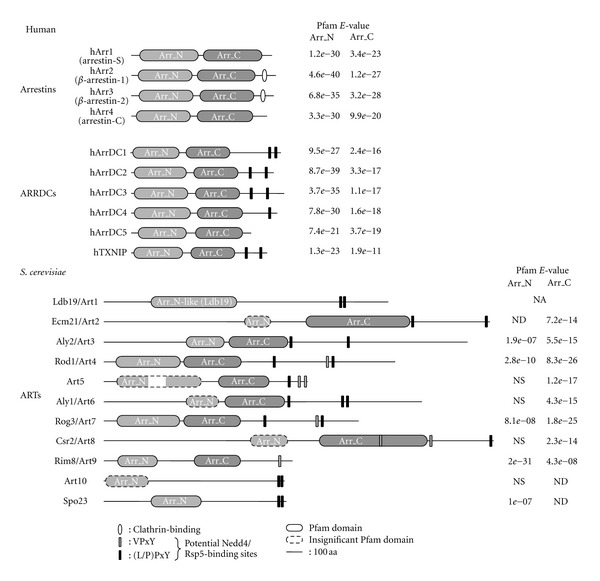
Schematic representation of the domain organization of human arrestins and arrestin-domain containing (ARRDC) proteins, and yeast ARTs (arrestin-related trafficking adaptors). Domains detected by Pfam 26.0 (http://pfam.sanger.ac.uk/) are shown and correspond to the following Pfam-A accessions: Arr_N: PF00339, Arr_C: PF02752, Arr_N-like (Ldb19): PF13002, along with the corresponding E-values for each domain (NA: not applicable; ND: not detected; NS: not significant). A putative Arr_N domain in Ecm21/Art2 was identified by alignment with the presumed Arr_N domain of Csr2/Art8. Clathrin-binding sites are depicted on *β*-arrestins; potential binding sites for ubiquitin ligases of the Nedd4 family are also indicated on arrestin-related proteins.

Visual and *β*-arrestins share a similar structure, with an arrestin fold in their N-terminal domains and a C-terminal tail [75–80]. It was proposed that visual and *β*-arrestins actually originate from an ancestral arrestin family from which they diverged relatively recently [25]. This ancestral family would also have given rise to proteins whose expression is not limited to metazoans: members of the Vps26 family, which display an arrestin-like fold [81, 82], as well as arrestin-related proteins (also coined *α*-arrestins) [25]. Indeed, proteins displaying sequence homologies to arrestins were first identified in the filamentous fungus *Aspergillus nidulans*, named CreD [83] and PalF [31], and more recent work in yeast allowed to identify additional members of this protein family renamed “ART” (arrestin-related trafficking adaptors) [34, 84, 85] that will be discussed later in this paper. In human, the ART family is composed of six members, named arrestin-domain containing 1–5 (ARRDC1–5) and TXNIP (Thioredoxin-interacting protein) (Figure 1). Therefore, arrestin-related proteins are expressed in all eukaryotes, except plants, which interestingly do not harbor Nedd4-like genes either [25].

### 4.1. Arrestin-Related Proteins as Endocytic Adaptors

A main difference between visual/*β*-arrestins and arrestin-related proteins is that the latter possess PPxY motifs (Figure 1). In agreement with the reported function of these motifs (see above), many studies have documented the ability of yeast arrestin-related proteins to interact with the only Nedd4-like ubiquitin ligase in *S. cerevisiae*, named Rsp5 [33, 34, 85–90]. Rsp5 is critical for ubiquitin-dependent intracellular trafficking pathways, such as endocytosis and MVB sorting [91]. However, most of the transporters lack PPxY motifs, and until recently, the molecular basis for the interaction between Rsp5 and transporters was unknown. It has become clear that yeast arrestin-related proteins fulfill this function, by acting as Rsp5 adaptors to mediate ubiquitylation and subsequent endocytosis of transporters [33, 34, 84, 85, 90]. Using a chemical-genetic screen, Emr and colleagues have identified Ldb19/Art1 as a regulator of the endocytosis of Can1, an arginine transporter [34]. The function of Ldb19/Art1 was also extended to the endocytosis of other amino acid transporters. In a parallel study, Nikko et al. showed that two other arrestin-related proteins, named Ecm21/Art2 and Csr2/Art8, are specifically involved in the downregulation of the manganese transporter Smf1 [85]. Altogether, around 10 arrestin-related proteins were identified in yeast [34, 84, 85], and gathered in a family referred to as “ART” (arrestin-related trafficking adaptors).

Contrary to the situation in mammalian cells, where *β*-arrestins mainly act at a late step in cargo sorting, studies in yeast suggested a role for arrestin-related proteins in cargo internalization at the plasma membrane [27]. Indeed, several yeast ARTs are involved in the signal-induced internalization of transporters in response to specific environmental signals [33, 34, 84, 85, 90]. In addition, Art1 relocalizes to the plasma membrane in response to the signal that induces amino acid transporter endocytosis [34, 92].

However, as for *β*-arrestins, the situation is probably more complex, and the role of ARTs in endocytosis may not be restricted to the plasma membrane. In the course of their study of the high-affinity iron-uptake protein complex Fet3/Ftr1 in yeast, Burd and colleagues documented an example of ubiquitin-independent internalization [43]. The results show that a nonubiquitylatable form of Fet3/Ftr1 can still be internalized but is constitutively recycled back to the plasma membrane, leading to an apparent defect in internalization. Although the involvement of an arrestin remains to be determined, it strongly suggests that in this system, cargo ubiquitylation by Rsp5 is required at endosomal compartments, rather than at the plasma membrane. In addition, two yeast arrestin-related proteins, Aly1/Art6 and Aly2/Art3, have been shown to localize to intracellular compartments and to control the trafficking of the general amino-acids transporter Gap1 between trans-Golgi and endosomes [93]. Consistent with these findings, Aly1 and Aly2 interact with both clathrin and Golgi-specific clathrin adaptor complex AP-1, thus suggesting that arrestin related proteins, as *β*-arrestins, promote clathrin-coat assembly and cargo targeting to clathrin-coated vesicles. Therefore, futures studies will be necessary to precise where yeast arrestin-related proteins act on cargo trafficking. Regarding their intracellular localizations, we can already hypothesize several modes of action within the ART family of proteins.

Like their yeast homologs, several human ARRDC proteins are able to interact with ubiquitin ligases of the Nedd4 family [94–97]. Among those, ARRDC3 was isolated in a screen designed to identify proteins involved in *β*2-AR ubiquitylation and degradation after agonist treatment [95]. ARRDC3, as *β*-Arr2 [24], was shown to bridge the interaction between Nedd4 and *β*2-AR, leading to the intriguing possibility that arrestin-related proteins might coordinate, together with *β*-arrestins, receptor ubiquitylation and degradation. Because both classes of arrestins have the ability to dimerize [94, 98], this raises the possibility of potential heterooligomers between arrestin and arrestin-related proteins that could reveal a complementary role between arrestin classes.

The basic function of arrestin-related proteins as ubiquitin ligase adaptor therefore seems strongly conserved. Of note, a role for ARRDC3 in the degradation of a cell surface adhesion molecule, integrin *β*4, was also pointed out, but its role as ubiquitin ligase adaptor in this context has not been investigated [99]. Future studies will indubitably unravel new connections between ubiquitin and arrestin-related proteins.

### 4.2. Other Functions of Arrestin-Related Proteins: An ESCRT Connection

As previously mentioned, arrestin-related proteins were initially identified in *A. nidulans* and named CreD [83] and PalF [31]. Interestingly, in both cases, a connection with the ubiquitin pathway was established. CreD was shown to interact physically with the Nedd4 homologue in *A. nidulans*, HulA, whereas PalF was found to be ubiquitylated in vivo. PalF, a protein involved in the ambient pH signaling in fungi, binds to the seven-transmembrane and putative pH sensor, PalH. This pointed out to many similarities between mammalian *β*-arrestins and this arrestin-related protein [31].

As in *A. nidulans*, the yeast PalF homologue Rim8/Art9 is essential for the proteolytic activation of the pH-responsive transcription factor, Rim101, in response to neutral-alkaline pH [100]. Interestingly, there is an intricate connection between the ESCRT machinery, involved in ubiquitin-dependent cargo sorting at the MVB, and this signaling pathway [100–102]. The ART Rim8/Art9 is central to the coordination of the ESCRT machinery and the pH-signaling pathway, as it interacts with both the putative pH sensor Rim21 and the ESCRT-I subunit Vps23 [89]. ESCRT appears to provide a platform for recruitment of a protein complex containing the ESCRT-III binding protein and ALIX homologue Rim20, that enables the proteolytic activation of the Rim101 transcription factor in response to the pH signal. Although initial studies suggested that this process takes place at the endosomal membrane [103], subsequent work supported the idea that arrestin-mediated recruitment of ESCRT in the fungal ambient pH signaling pathway may occur at the plasma membrane [89, 104]. Similarly, some of the human ARRDCs interact with the Vps23 homologue TSG101 or the ESCRT-associated protein ALIX [90, 96]. In particular, ARRDC1-mediated recruitment of ESCRT appears to drive the formation of microvesicles at the plasma membrane that may be involved in intercellular communication [94, 105]. Interestingly, this situation is reminiscent of the budding step of different enveloped RNA viruses, which recruit ESCRT components through similar interactions to promote membrane scission and subsequent viral particle release [106]. Accordingly, overexpression of several ARRDCs inhibits murine leukemia virus (MLV) viral particle release in a PPxY-specific way [96]. Therefore, ARRDCs may act as adaptors between Nedd4-like enzymes and the ESCRT machinery, in viral budding.

Finally, the connection between arrestin and the ESCRT machinery may not be restricted to arrestin-related proteins. Indeed, and as previously mentioned, *β*-Arr1 was found to interact with STAM-1 (signal-transducing adaptor molecule), a component of ESCRT-0, to regulate endosomal sorting of CXCR4 [69].

## 5. Regulation of Arrestin Function by Ubiquitin

### 5.1. Regulation of *β*-Arrestins by Ubiquitylation

Arrestins are specifically recruited to the cargoes following agonist stimulation (receptor) or in response to the presence of the substrate (transporter, channel), thus suggesting that they are regulated to mediate an adapted response of the cell to extracellular changes.

As mentioned previously, Shenoy and colleagues showed in a seminal article that *β*-Arr2 is itself ubiquitylated in response to agonist treatment [30]. Ubiquitylation of *β*-Arr2, in contrast to that of *β*2-AR, does not require Nedd4, but an ubiquitin ligase of the RING family, Mdm2. As this modification occurred upon stimulation, this suggested a role for *β*-Arr2 ubiquitylation in *β*-AR trafficking. Indeed, Mdm2 knockdown caused a defect in *β*2-AR internalization. Thus, *β*-Arr2 ubiquitylation appears to play a key role in GPCR trafficking, and several lines of evidences support this idea.

To address directly the importance of this posttranslational modification and to avoid potential indirect effects of the knockdown of the Mdm2 ubiquitin ligase on *β*2-AR trafficking, studies were performed using both a nonubiquitylatable mutant form of *β*-Arr2 (*β* − *Arr*2^0 K^) and a translational fusion of ubiquitin to *β*-Arr2 (*β*-Arr2-Ub) [107]. These experiments showed that *β* − *Arr*2^0 K^ recruitment to the plasma membrane was only transient and unable to trigger internalization of *β*2-AR. On the opposite, translational fusion of ubiquitin to *β*-Arr2 led to its co-trafficking with *β*2-AR into endosomal compartments [107]. Previous observations had classified GPCRs in two classes (A and B), based on the interaction pattern between receptor and *β*-arrestin. Interaction of *β*-arrestin with class A receptors (e.g., *β*2-AR) only takes place at the plasma membrane, while its interaction with class B receptors (e.g., angiotensin II type 1a receptor: AT1aR, or vasopressin V2 receptor: V2R) is more stable and persists even after receptor internalization [108]. Interestingly, the increased stability of the interaction between class B receptors and *β*-arrestin correlates with a sustained *β*-arrestin ubiquitylation, which is not observed with class A receptors [36]. Indeed, even if  *β* − *Arr*2^K0^ is able to interact with the receptor* in vitro*, this interaction is weaker than that displayed with the wild type form *in vivo*. On the opposite, translational fusion of ubiquitin to *β*-Arr2 displays a stronger binding than wild type *β*-Arr2 [107]. *β*-Arr2 ubiquitylation therefore appears to reinforce the interaction with *β*2-AR.

Because *β*-Arr2 is capable of interacting with the endocytic machinery, such as clathrin or clathrin adaptors, the failure of  *β* − *Arr*2^K0^ to promote *β*2-AR internalization could originate from an impaired interaction with these components. Indeed,  *β* − *Arr*2^K0^ exhibits a weaker interaction with clathrin than the wild-type form [107]. While clathrin is not known to interact with ubiquitin, *β*-Arr2 ubiquitylation might stabilize the interaction with clathrin through other components of the endocytic machinery such as Eps15/epsin proteins that are able to bind both ubiquitin and clathrin.

*β*-Arr2 ubiquitylation was also shown to affect its scaffolding function for signaling proteins. The amplitude of *β*-arrestin-mediated activation of ERK (extracellular signal-regulated kinase) correlates with the *β*-Arr2 ubiquitylation status. Although *β*-Arr2 ubiquitylation was not required for its interaction with MAP kinases (such as c-Raf and ERK), translational fusion of ubiquitin to *β*-Arr2 led to an increased level of ERK activity in endosome localized receptor complexes (signalosomes). Consistent with these findings, *β*-arrestin ubiquitylation promote its association with membrane. Again, ubiquitylation appears to function in stabilizing the *β*-arrestin-mediated interaction between the receptor and signaling proteins [107].

Finally, arrestins undergo conformational changes upon binding to activated receptors [109]. Ubiquitin modification could therefore contribute to the proper rearrangement of the *β*-arrestin structure, leading to optimal interactions with its partners, and this awaits further investigations.

### 5.2. Regulation of Arrestin-Related Protein by Ubiquitylation

Many arrestin-related proteins have also been reported as substrates for ubiquitylation, both in fungi and human [31, 33, 34, 89, 90, 94, 96, 97, 110]. Ubiquitylation of these proteins, in contrast to that of *β*-arrestins, is triggered by ubiquitin ligases of the Nedd4 family. Therefore, arrestin-related proteins are adaptors as well as targets of the same ubiquitin ligases.

The yeast arrestin-related protein Ldb19/Art1 is required for the endocytosis of amino acid permeases, such as the arginine transporter, Can1. Failure to endocytose Can1 leads to sensitivity of the cells to canavanine, a toxic analog of arginine. A nonubiquitylatable mutant of Ldb19/Art1 cannot grow on this drug, suggesting that Can1 remains at the plasma and therefore that Art1 is not functional [34]. The importance of ubiquitylation for ART function was also demonstrated for Rod1/Art4, involved in the glucose-induced endocytosis of carbon sources transporters [33, 84]. Rod1/Art4 is ubiquitylated in response to glucose exposure and a nonubiquitylatable mutant is unable to promote the endocytosis of the lactate transporter, Jen1, following glucose treatment [33]. Altogether, these data indicate that ART ubiquitylation is crucial for their function in endocytosis. Human arrestin-related protein ARRDC3 was isolated in a screen designed to identify genes involved in *β*2-AR degradation, and acts as a Nedd4 adaptor for *β*2-AR ubiquitylation [95]. While ARRDC3 ubiquitylation has not yet been observed, ARRDC1 and TXNIP were shown to be ubiquitylated by ubiquitin ligases of the Nedd4 family [90, 96, 97]. Thus, it is tempting to speculate that the same regulation applies in fungi and human.

The ubiquitylation of the arrestin-related protein PalF in *A. nidulans* is triggered in a signal-(alkaline pH) and receptor-(PalH) dependent manner [31]. PalF ubiquitylation appears as a major determinant of its activity, since the translational fusion of ubiquitin to PalF leads to a constitutive activation of the pathway [110]. The yeast PalF homologue Rim8/Art9 was shown to be monoubiquitylated [89]. Monoubiquitylation of Rim8/Art9 occurs on a lysine residue in its C-terminus and, as for all other ARTs described to date in yeast, is performed by Rsp5, which binds to a PxY motif near the ubiquitylation site. This monoubiquitylated residue, together with a SxP motif, contributes to the interaction of Rim8/Art9 with the ESCRT-I subunit Vps23 via its ubiquitin-binding domain, UEV (ubiquitin E2 variant) [89]. Interestingly, Vps23 binding appears to control the levels of monoubiquitylated Rim8/Art9, thus suggesting that this interaction either promotes Rim8 ubiquitylation or prevents its further polyubiquitylation and possibly its degradation. Interaction of human ARRDC1 with the Vps23 homologue Tsg101 was shown to be mediated by a PSAP motif which, like the SxP motif in Rim8, is located at the protein C-terminus [94, 96]. In addition, it was proposed that the ubiquitylation of ARRDC1 is important for its function [94]. However, this is based on results obtained upon depletion of the corresponding Nedd4-like ligase WWP1, which in principle could also impair a potential adaptor function and may have off-target effects. Therefore, the identification and mutation of the ubiquitylation sites will be critical to address this question.

### 5.3. Dynamic Regulation of Arrestin Ubiquitylation

*Phosphorylation-Dependent Ubiquitylation? *Because ubiquitin ligases target a large number of proteins in the cell, their activity toward a given substrate is usually indirectly regulated through substrate accessibility, either by the use of adaptor proteins, or by post-translational modification of the substrate, such as phosphorylation [111].

Interestingly, cytosolic *β*-arrestins are constitutively phosphorylated, and undergo dephosphorylation upon binding to the activated receptor. *β*-Arr1 dephosphorylation is required for *β*2-AR internalization, but not for its desensitization [112]. Indeed, a *β*-Arr1 mutant mimicking constitutive phosphorylation displays a weaker interaction with clathrin but an unaltered *β*2-AR binding [112]. Similar data were reported for *β*-Arr2, and the phosphorylation site was localized near the clathrin and AP-2 binding motifs, thus providing an explanation as to why *β*-Arr2 phosphorylation regulates the interaction with clathrin/AP-2 [113]. Additionally, the phosphorylation of the major visual arrestin in *Drosophila* (Arr2) also regulates its interaction with clathrin [114].

Importantly, once the receptor is internalized, *β*-Arr1 is rephosphorylated. These dynamic phosphorylation/dephosphorylation events suggest the involvement of kinases and phosphatases whose activation is coordinated in response to agonist exposure. Interestingly, *β*-Arr1 is phosphorylated *in vitro *by ERK kinases and accordingly, the modulation of ERK activity *in vivo* affects *β*-Arr1 phosphorylation, thus providing an inhibitory feedback control of its function [115].

Although *β*-arrestins are both dephosphorylated and ubiquitylated upon receptor binding, an eventual relationship between these two modifications remained to be addressed. Such a link has been described for the yeast arrestin-related protein Rod1/Art4, involved in the glucose-induced endocytosis of carbon sources transporters [33]. As for *β*-arrestins, Rod1/Art4 dephosphorylation and ubiquitylation occurs in response to an external signal-in this case, glucose. The yeast homologue of AMPK (5^'^-AMP-activated protein kinase), Snf1, and its counteracting phosphatase PP1 (protein phosphatase 1) control the phosphorylation status of Art4/Rod1 in response to glucose availability. Therefore, in the absence of glucose, Art4/Rod1 is phosphorylated and endocytosis is inhibited. This inhibitory effect results from the ability of phosphorylated Art4/Rod1 to bind 14-3-3 proteins, thereby hindering its ubiquitylation by Rsp5 and hence preventing its activation [33].

Interestingly, phosphorylation of another yeast arrestin-related protein, Ldb19/Art1, also regulates its function. A recent study indicated that Ldb19/Art1 is subject to phosphoinhibition through the action of the TOR (target of rapamycin) effector and protein kinase Npr1, thus allowing cells to regulate amino acid transporter endocytosis in response to the nitrogen status [92]. While the overall phospho-inhibition mechanism recalls that of Rod1/Art4, Ldb19/Art1 ubiquitylation is uncoupled from its phosphorylation, suggesting a different regulatory mechanism [34]. The identification of Npr1-dependent phosphorylation sites on Ldb19/Art1 allowed generating a nonphosphorylatable mutant form of the protein. Interestingly, this mutant fails to be translocated at the plasma membrane upon stimulation, which likely explains why transporters endocytosis is impaired [92]. Further work will be needed to understand the molecular mechanism of this phosphorylation-dependent inhibition.

Other examples of arrestin-related proteins that are subjected to phosphorylation which include *A. nidulans* PalF, involved in ambient pH sensing, although the regulatory mechanism appears to be different since PalF undergoes phosphorylation instead of dephosphorylation, in response to the ambient pH signal [31]. The same result has been observed in the pathogenic yeast *Candida albicans*, where the PalF homologue Rim8 is also phosphorylated in response to neutral-alkaline pH [116]. Although phosphorylation of Rim8/Art9 in baker's yeast has not been reported, its ubiquitylation does not appear to be regulated by ambient pH, in contrast to that of PalF, which is induced by alkaline pH [89]. The apparent lack of regulation of Rim8/Art9 ubiquitylation is consistent with its role in Vps23 binding, which appears to occur even in nonstimulated conditions. Thus, the pH-dependent regulation of PalF ubiquitylation in *A. nidulans* may reflect an additional level of regulation in this organism.

From these studies, it emerges that arrestin-related proteins are often modified posttranslationally in response to stimulation, either by phosphorylation, ubiquitylation, or both. A crosstalk between phosphorylation and ubiquitylation has been evidenced. How these modifications operate to coordinate arrestin function is unknown, and this provides new avenues for research in this field.

*Ubiquitylation and deubiquitylation. *Ubiquitylation, akin to phosphorylation, is a reversible process. Therefore, deubiquitylation appeared as a possible mechanism for regulation of arrestin function. In support of this idea, the transient *β*-Arr2 ubiquitylation associated to class A receptor suggested that deubiquitylation occurs rapidly after agonist stimulation. Indeed, the ubiquitin-specific protease 33 (USP33) was shown to deubiquitylate *β*-Arr2 following *β*2-AR binding [35]. USP33 knock-down led to an increase in *β*-Arr2 ubiquitylation. This was accompanied by a stronger interaction with the receptor, and a prolonged *β*-Arr2-dependent MAP kinase signaling. These findings are consistent with the phenotypes of cells expressing a translational fusion of ubiquitin to *β*-Arr2 (see above) and provide an additional mechanism for the regulation of arrestin function. Strikingly, recruitment of USP33 to *β*-Arr2 depends on the receptor class, in agreement with previous finding that receptor class determines the kinetics of *β*-Arr2 deubiquitylation [36]. In addition, receptors belonging to the same class can target different lysine residues on *β*-Arr2 for ubiquitylation [32]. Therefore, binding of *β*-Arr2 to different receptors may trigger distinct conformational change that could modulate both ubiquitylation sites accessibility and association with deubiquitylating enzymes, hence leading to a different functional output—in full support of the concept of an “ubiquitylation code.”

## 6. Concluding Remarks

In this paper, we emphasized the many relationships between ubiquitin metabolism and arrestin biology. Many proteins of the arrestin family act as ubiquitin ligase adaptors and are required for ubiquitylation of endocytic cargo. In particular, recent data obtained on arrestin-related proteins have pointed out several features shared with *β*-arrestins, such as the intimate connection existing between these proteins and ubiquitin ligases of the Nedd4 family. In addition, ubiquitin also regulates arrestin function in a yet undefined way, which is now critical to understand. However, the emergence of new regulatory mechanisms involving a now expanded family of arrestin proteins, combined with the multiplicity of model organisms, is likely to favor the rapid evolution of concepts in arrestin biology.

## Abbreviations

AIP4: Atrophin-interacting protein 4
AP-1/AP-2: Clathrin adaptor protein
ARRDC: Arrestin-domain Containing
ART: Arrestin-related trafficking adaptors
*β*-Arr: *β*-arrestin
*β*2-AR: *β*2-adrenergic receptor
ERK: Extracellular signal-regulated Kinase
ESCRT: Endosomal sorting complex required for transport
GPCRS: G-protein-coupled receptors.
MAP: Mitogen-activated protein
MVBs: Multivesicular bodies
Nedd4: Neural precursor cell expressed developmentally downregulated protein 4
RING: Really interesting new gene
TXNIP: Thioredoxin-interacting protein.
